# Epidemiological trends in community acquired acute Kidney Injury in Pakistan: 25 years Experience from a Tertiary Care Renal Unit

**DOI:** 10.12669/pjms.37.2.3876

**Published:** 2021

**Authors:** Rubina Naqvi

**Affiliations:** 1 Prof. Rubina Naqvi, MBBS; MD (Nephrology), FISN, PGD Bioethics. Department of Nephrology, Sindh Institute of Urology and Transplantation (SIUT), Civil Hospital, Karachi, Pakistan

**Keywords:** Acute Kidney Injury (AKI), Epidemiology, Community acquired, Medical AKI, Obstetrical AKI

## Abstract

**Background:**

Epidemiological studies of community acquired acute kidney injury (AKI) are sparse especially from South Asia and none has published from Pakistan. Reported incidences from different countries vary with use of different criteria of defining AKI. There is also variation found in different class of income countries, hospital based versus community based AKI.

**Methods:**

The current study was carried out in all adult AKI patients developing community acquired AKI and coming to a tertiary care renal institution from January 1990 to December 2014. This is a retrospective data collection from patient’s records and AKI was defined according to KDIGO guidelines. Trends among different groups which are classified in medical, obstetrical and surgical were observed and presented.

**Results:**

In medical AKI there has been found a rise in toxic rhabdomyolysis, vivax malaria and dengue infection during later part of study. In obstetrical AKI observed continuous rise in numbers contributing to total AKI during these years. Surgical AKI included obstructed cases during initial ten years and only surgical trauma during later 15 years. Older age on presentation in medical AKI, and thrombocytopenia, deranged coagulation, deranged liver function, hyperkalemia, requirement of mechanical ventilation and multi organ failure in all groups remained predictors of higher mortality.

**Conclusion:**

From Pakistan epidemiology for community acquired AKI has never been published on a large scale and this study would remain source of great information in this regard over coming years.

## INTRODUCTION

Acute kidney injury (AKI) is universally acknowledged to be a common, serious and under-recognized condition. It can occur in a variety of clinical settings, and is a predictor of adverse outcomes in the short and long-term. It is estimated that approximately 13.3 million people develop AKI around the world, of which 1.7 million die, mostly in Lower Income (LIC) and Lower Middle Income countries (LMIC).^1^

AKI which is one of most prominent subject in nephrology, its incidence and prevalence varies widely world over. The main reason for such wide variation remained widely used definitions for AKI which include an abrupt and sustained decline in renal function, a temporary rise in creatinine to at least 300 μmol/L, an increase in serum creatinine of >0.5 mg per deciliter over the baseline to an increase of >50% over the baseline value, to addition of RIFLE criteria in May 2002 in a meeting in Vicenza, AKIN classification in a meeting in Amsterdam in September 2005 and later KDIGO guidelines laid out in 2012 for definition of AKI.^2^

Over time, it has become clear that in high income countries, AKI develops primarily in already hospitalized patients, often with multiple pre-existing conditions. However, the AKI population in low and middle income countries is generally persons who were previously healthy. A complex interplay between geographical characteristics, environmental factors, and poverty drives the presentation and outcome of this condition.^3^,^4^ AKI has a huge impact on families, societies and national economies. Knowledge of the diversity of AKI is relevant to developing a global response to mitigate the impact of this health condition, especially in resource-poor settings.

Pakistan, with a population of 215 million is a low-middle income country (nominal per capita GDP US$1,357 in 2019) and has relatively poor health development indicators. According to the United Nations Human Development Report, Pakistan ranks 142 out of 177 countries.^5^ The population growth rate in Pakistan has been reported 1.92% and Health expenditure is 0.919% of GDP.^6^

Knowledge of epidemiology is essential to reduce preventable mortality due to AKI, as articulated by the ISN’s 0by25 program. In a country like Pakistan where almost two-thirds of the population resides in rural areas,^7^ many people with AKI cannot reach renal units, making it practically impossible to derive a community-based estimate of the incidence or prevalence of this condition.

The present study describes the evolution of the pattern of community-acquired AKI over a 25-year period as seen at a large tertiary care hospital in Pakistan.

## METHODS

This is a retrospective cohort study carried out on the basis of chart review of all patients with diagnosis of AKI admitted from January 1990 to December 2014, to the Sindh Institute of Urology and Transplantation (SIUT) Pakistan. SIUT located in city of Karachi and currently has satellite center within city and other cities; is semi-government tertiary renal care hospital funded partially by government and mainly by philanthropists; SIUT provides all facilities to patients free of cost. Since care is frequently life long, the setup provides the opportunity of gathering large amounts of data and long term follow up.

In 1990 SIUT had approximately 36 beds; by 2014 this number had increased to 450. With its model of free care to all patients, SIUT draws patients from all over the country (travel distance may extend to 1,550 Km) and sometimes from neighboring countries lacking medical facilities like Afghanistan. Patients with AKI are admitted and provided appropriate care including dialysis until death, (renal transplant from related donors in case they developed end stage renal failure) or recovery trends in renal function. Patients are discharged once it is assumed that they do not require regular dialysis and are asked to follow up in the outpatient clinics. Since many patients belong to poor socio-economic backgrounds and come from far-flung locations, follow up remains incomplete in many patients unless they experience compelling symptoms that forces them to seek medical care again.

AKI in this study was defined according to KDIGO guidelines:^2^ increase in serum creatinine concentration or decline in urine output were used to classify patients as having AKI. Although these patients were treated before the KDIGO criteria, it was applied retrospectively to a group of patients while going through history and presentation in records. Patients with past co-morbidities which can impact GFR like diabetes mellitus and long standing hypertension were excluded from the study.

We reviewed the records and extracted information on patients’ demographic characteristics, cause of AKI, treatment provided and outcomes. The parameters recorded from day of hospital admission are given in Table-I along with serological tests were done in selected cases, use of parenteral fluids before renal replacement therapy, renal replacement therapy, sessions of hemodialysis or days on peritoneal dialysis, renal biopsy and outcome.

**Table-I:** Demographics of all patients.

*Parameters*	*Mean ±SD*	*Median*	*Inter quartile range*
***Age (years)***			
Medical	35.91±15.81	32	22-45
Obstetrical	27.83±5.66	27	24-31
Surgical	43.24± 16.746	42	30-55
***Duration of Insult (days)***			
Medical	10.32±7.02	8	6-14
Obstetrical	8.92±6.44	7	4-12
Surgical	9.62± 6.48	7	5-13
***Hemoglobin(G/dL)***			
Medical	10.52± 3.16	11	8-13
Obstetrical	8.14± 2.06	8	7-10
Surgical	10.51± 2.67	10.7	8.7-12.5
***WCC x10^3^/µl***			
Medical	14.99± 10.65	13	9-18
Obstetrical	18.99± 9.53	17	12-23
Surgical	16.53± 9.54	13.9	10-21
***Platelet x10^3^/µl***			
Medical	221.18± 155.50	200	110-299
Obstetrical	221.11± 173.30	169	97-306
Surgical	238.07± 146.34	210	135-315
***Urea mg/dl***			
Medical	262.05± 116.42	245	178-324
Obstetrical	213.38± 99.39	197	139-265
Surgical	251.97± 107.39	233	168-313
***Creatinine mg/dl***			
Medical	10.45± 5.35	9	7-13
Obstetrical	9.79± 4.62	9	7-12
Surgical	9.36± 4.48	8.41	6.3-11.49
***Sodium mEq/L***			
Medical	131.89± 8.82	132	127-137
Obstetrical	134.07± 8.82	135	129-139
Surgical	131.77± 9.73	132	126-138
***Potassium mEq/L***			
Medical	4.54 ±1.38	4	4-5
Obstetrical	4.96± 1.24	5	4-6
Surgical	4.61± 1.30	4.6	3.6-5.5
***Bicarb mEq/L***			
Medical	14.27± 5.40	14	11-18
Obstetrical	14.67± 4.89	14	11-18
Surgical	15.51± 4.82	15	12-18
***AST U/L***			
Medical	284.58± 905.67	56	30-136
Obstetrical	200.41± 512.21	50	25-139.25
Surgical	194.23 ±507.56	52	31-119
***ALT U/L***			
Medical	152.70± 424.32	40	23-84
Obstetrical	163.31± 505.96	39	19-109
Surgical	200.80 ±685.06	41	25-96
***Alk Phos U/L***			
Medical	137.86± 119.41	103	72-161
Obstetrical	165.34± 135.61	128	94-187
Surgical	167.97± 158.30	122	93-187.25
***LDH U/L***			
Medical	1677.95± 2523.34	973	447-1815
Obstetrical	1735.21± 1499.89	1338	675-2220
Surgical	1864.91± 1803.77	1337.50	622.75-2304.00
CPK* U/L			
Medical	30777.59 ±129994.081	1521	324-9388
Obstetrical	-	-	-
Surgical	3670.82± 5858.32	1532.50	319.75-4274.00

* done in selected cases

The outcomes were divided into death, complete recovery, when patients were followed up till gaining normal kidney functions; partial recovery, when patients were off dialysis but were lost to follow up within a 90-day period, and as having developed chronic kidney disease (CKD) when the eGFR was documented to be <60 ml/min/1.73m2 beyond 90 days and end stage renal failure (ESRF) when never became dialysis free.

The studied population has been divided over four time periods - the first being from January 1990 to December 1999, followed by three five year periods. The study protocol was in accordance with the Declaration of Helsinki and SIUT’s Institutional Ethical Review Committee has granted permission for publishing this data.

### Statistical Analysis

Statistical analysis was done on SPSS version 20.0. Continuous variables were expressed as mean ± standard deviation or median with inter-quartile range. Frequencies and percentages were computed for categorical variables. Independent “t” test was used to determine the mean difference between outcomes while Chi- square independent or Fisher’s Exact test were used to determine the proportions difference between outcomes with different presenting symptoms and multivariate logistic regression was done to identify the independent risk factors. P value <0.05 was considered as significant.

## RESULTS

A total of 5,623 patients were diagnosed to have AKI during the study period of 1990-2014. The demographic characteristics of the population are shown in Table-I. The average age of the population was 37.74±14.89 (range 18-100) years, and a total of 2,851 were male and 2,772 were female (M:F 1.02:1). Excluding those with AKI due to obstetric causes, males constituted 68% of the population.

A breakup of the medical causes of AKI is shown in Table-II. The most striking change was the emergence of poisoning due to Paraphenylene diamine (PPD) causing toxic rhabdomyolysis, and infection due to dengue in the later time periods. Rare causes or patients with biopsy findings of acute tubular necrosis with no supporting history are included in the miscellaneous group, while in 239 cases causes for AKI still remained unknown as they could not be biopsied.

**Table-II:** Medical Causes of AKI.

*Causes*	*Group I (1990-1999)*	*Group II (2000 - 2004)*	*Group III (2005 - 2009)*	*Group IV (2010 - 2014)*	*Total 25 years (n=3,389)*
Acute GE	302	190	183	178	853
Malaria	124	93	203	251	671
Rhabdomyolysis	67	35	52	180	334
GN/ Vasculitis	88	32	36	80	236
Sepsis	71	31	28	32	162
Nephrotoxic Drugs	74	24	24	33	155
Snake Bite	38	16	30	31	115
Poisons	13	8	11	15	47
HUS	18	8	6	13	45
Dengue	0	2	9	32	43
Hepato Renal Syndrome	11	3	6	16	36
Multiple Myeloma	12	5	5	10	32
Cardio Renal Syndrome	10	4	5	15	34
Acute Pancreatitis	6	3	4	6	19
Scorpion Sting	1	2	4	11	18
Misc. Causes	86	70	90	104	350
Unknown Causes	42	53	55	89	239

The different causes alone or in combination in obstetrical or pregnancy related AKI are listed in Table-III. There have been continuous rising trends in pregnancy related AKI: during the initial 10 years of our data, pregnancy related AKI was 21.64% of the total. In the last 5 years, this figure jumped to 35.27%.

**Table-III:** Obstetric Causes of AKI.

Causes	Group I (1990-1999) N=357	Group II (2000 - 2004) N= 163	Group III (2005- 2009) N=286	Group IV (2010 - 2014) N=635	Total 25 years N=1441
Abortion	49 (13.7)	22 (13.49)	35 (12.2)	39 (6.1)	145
Ante Partum Haemorhage*	113	50	83	180	426
Eclampsia/PET*	66	17	37	107	227
Intra Uterine Death*	145	76	70	291	582
Post Partum Haemorhage*	117	63	57	167	404
Sepsis*	60	21	51	69	201
HUS/HELLP	4 (1.1)	7 (4.2)	11 (3.84)	12 (1.88)	34

* more than one cause contributing to insult in many women

The overall mortality was 11-33% among different groups. Regarding the outcome of AKI, 53.35 percent showed a total recovery while a partial recovery was noted in 22.81 percent. Of those who died, 14.65 percent died during the acute phase of illness and 9.19 percent developed ESRF. Significantly higher morality was seen in patients presenting with the following issues: thrombocytopenia, hyperkalemia, deranged liver function, coagulopathy, and need for mechanical ventilatory support or multi-organ failure (MOF). Altered Glasgow Coma Scale (GCS) and older age were significantly associated with mortality in the medical group but not in others (Table-V).

**Table-IV:** Surgical Causes of AKI.

Causes	*Group I (1990 -1999)*	*Group II (2000 - 2004)*	*Group III (2005 - 2009)*	*Group IV (2010 - 2014)*	*Total 25 years (n=793)*
Surgical Trauma	135	48	74	87	344
Obstructed	449	Not recorded	Not recorded	Not recorded	

**Table-V:** Predictors of mortality among different groups of AKI.

Factors	Groups	P value
Age	Medical	<0.001
	Obstetrical	NS
	Surgical	NS
Gender	Medical	NS
	Surgical	NS
Thrombocytopenia	Medical	<0.001
	Obstetrical	<0.001
	Surgical	<0.001
Thrombocytosis	Medical	NS
	Obstetrical	NS
	Surgical	NS
Hyponatremia	Medical	NS
	Obstetrical	NS
	Surgical	NS
Hyperkalemia	Medical	<0.001
	Obstetrical	<0.001
	Surgical	<0.001
Acidosis	Medical	NS
	Obstetrical	NS
	Surgical	NS
Deranged liver function	Medical	<0.001
	Obstetrical	<0.001
	Surgical	<0.001
Deranged coagulation	Medical	<0.001
	Obstetrical	<0.001
	Surgical	<0.001
Hypotension	Medical	NS
	Obstetrical	NS
	Surgical	NS
Abnormal GCS	Medical	<0.001
	Obstetrical	NS
	Surgical	NS
Requirement of mechanical ventilation	Medical	<0.001
	Obstetrical	<0.001
	Surgical	0.002
MOF	Medical	NS
	Obstetrical	0.002
	Surgical	0.002
Combination of hypotension and MV	Medical	<0.001
	Obstetrical	<0.001
	Surgical	NS
Combination of hypotension, MV and MOF	Medical	<0.001
	Obstetrical	<0.001
	Surgical	<0.001
Combination of abnormal GCS, hypotension and MV	Medical	0.004
	Obstetrical	0.002
	Surgical	<0.001

MV=Mechanical Ventilation, MOF=Multi Organ Failure, GCS= Glasgow Coma Scale

Statistically, the highest mortality in the medical group was seen in patients with acute pancreatitis, cardio renal syndrome, hepato renal syndrome, hemolytic uremic syndrome, malaria and poisons. In the surgical group, firearm injuries, road traffic accidents, multiple bone fractures and acute abdomen resulting from visceral perforation were significant causes resulting in death of patients in the acute phase (p value <0.05).

The overall requirement for renal replacement therapy was 82 percent, indicating the severity of kidney injury on arrival. Hemodialysis was the most common modality (%). Intermittent peritoneal dialysis was performed in 70 cases during 1990-99. CRRT was not done in any of the patients in this population. Diversion procedures like percutaneous nephrostomies or Double J stenting in bilateral obstructed kidney in Group-I patients were done.

## DISCUSSION

The epidemiology of AKI has been poorly described and scarcely reported from lower income and middle lower income countries. Community acquired AKI (CA-AKI) studies are few, especially from our part of the world. A multicenter epidemiological study looking from South-East Asia that looked at AKI in intensive care units (ICUs) reported occurrence of AKI in 52.9% of ICU patients. This was based on data collected from initial 28 days of ICU admission.^8^ A study published from Nepal which addresses all renal disorders in outpatients or those hospitalized under nephrology care, reports AKI in 2% of out patients and in 14% of hospitalized patients.^9^

A study from Bangladesh found 105 cases of CA-AKI over a period of four years. The study reported different causes of AKI but did not provide the number of total admissions to this hospital during this period.^10^ From Pakistan, a recent study that looked at ICU patients over six months revealed AKI in 68.5% of ICU patients.^11^ Variations in prevalence can also occur among critically ill, sepsis association, major surgeries or among trauma patients.

Etiologies of AKI also vary in accordance with geographic and socio-economic status. Differences can be found even within studies published from the same country. For example, a study published from Northern Pakistan reports AKI in 16-20 % of hospital admission and 7-10% of these were due to obstetrical causes,^12^ whereas a study published from a large tertiary care center in the South of the country reports 57 .44 % medical and 22.87 % obstetrical cases of AKI.^13^ Similarly, a study published from the North of India reports incidence of AKI to be 0.64% of hospital admissions, out of which 29% were related to use of nephrotoxic agents, followed by decreased renal perfusion 21%, major surgery 18% and sepsis 17%.^14^ A study from Eastern India reports medical causes contributing 68.3%, surgical 17.9% and obstetrical 13.9% to the incidence of AKI.^15^

The main focus of current study will remain on CA-AKI as this is a population based study carried out at a tertiary care renal unit in a LMIC, and discussion will mainly be diverted towards variation among likewise countries in this particular group of AKI.

CA-AKI from HICs has mainly been reported with rhabdomyolysis from infections, seizures, binge (prolonged lying) and disasters like earthquake or accidental trauma causing crush syndrome. Other causes include vascular events (like renal vein thrombosis) or drugs like NSAIDs.^16^ In the Tropics, CA-AKI has been reported from tropical infections like malaria, leptospirosis, dengue, scrub typhus, yellow fever, plants, herbicides, snake bite, scorpion stings and diarrheal illness.^17^ From Latin America, during 1957-1966, the most frequent causes of AKI were non compatible blood transfusion and surgery, whereas from 1980-1982, use of nephrotoxic agents was more common while in 1993, sepsis, congestive heart failure and hypotension were reported more frequently. Later, infections like leptospirosis and dengue and snake bite induced AKI and multiple bee stings have also seen from this region.^18^ In the present study non compatible blood transfusions and multiple bee stings are included in the miscellaneous group as their number were small thus outcomes could not be separately evaluated.

From South Africa, commonly reported causes include nephrotoxicity with use of herbal remedies in 1978, sepsis between 1986-1988, and HIV associated nephropathy in 2005-2006. From Sudan, infections like malaria and typhoid, PPD poisoning, snake bites, obstruction from urinary calculi and acute GN with descending frequency are reported. Morocco has reported sepsis, leptospirosis, toxic rhabdomyolysis followed by acute GN and obstruction as causes of AKI.^19^ In the current study, toxic rhabdomyolysis secondary to PPD ingestion was observed during the later period (Group IV). Dengue infection also emerged in the later part of the study, whereas malaria, GN and diarrhoeal illness showed little variation over different time periods (Table-II).

A study published from Bangladesh in past reported medical causes 78.33%, surgical 18.33% and obstetrical in 10.83% of CA-AKI and they have shown overall survival rate of 75%.^20^ Another more recent study from the same country reveals still lower contribution of obstetrical AKI 4% to the total CA-AKI. Here medical causes were contributing 86%.^10^

This institution, SIUT, has previously published studies that show malaria, dengue infections, rhabdomyolysis, snake bite, scorpion sting, and obstetrical reasons as causes for AKI.^21^-^26^

Among tropical infections other than malaria and dengue, leptospirosis and scrub typhus has also been reported contributing to CA-AKI^27^ but somehow we did not find these two in our series, leptospirosis was suspected in some cases and serological tests done but only one result revealed very minimally raised titer for leptospirosis with the rest clearly negative. Other studies from the country were searched and a published study from veterinary surgeons reported leptospirosis in 40.83 % of people handling infected animals but this study did not highlight renal injury in these people.^28^ Studies from Nepal and Bangladesh^9^,^10^ have also not reported this infection in their AKI experience, but Indian studies have highlighted cases reported from leptospirosis.^17^ Similarly, we were unable to find any scrub typhus causing AKI in the present series but we had a few enteric fever cases which were included in the miscellaneous group.

Trends of obstetrical AKI in our studies remain on the rise,^24^ while our neighboring country India has shown declining trends in obstetrical AKI.^29^ One possible reason for this decline could be the legalization of abortion, resulting in a decrease in septic abortions and related complications. Another possibility for decline in complicated obstetrics in India is launching a program which offers cash rewards to women who gave birth to their children in healthcare facilities.^30^ After this provision, there has been a substantial increase in the number of women delivering in institutions. In present study in the obstetrical category many women had more than one insult contributing to the cause for AKI, for example, abruptio placentae with concealed or revealed hemorrhage can cause intrauterine fetal death (IUD) and may require emergency lower segment caesarean section (LSCS) and poor handling at peripheral center may add sepsis to all this. IUD in combination of pre eclampsia (PET) can be explained on basis of widespread vascular endothelial cell dysfunction resulting in vasospasm, inadequate placental circulation. 31 Some studies have reported IUD as independent risk factor for maternal disease.^32^ Similarly LSCS alone has also been reported to cause maternal morbidity and contribute to AKI.^33^ Among all three categories in our study, we had the highest number of partially recovered patients in the obstetrical group which may highlight the socio cultural setup of the country and gender biases that result in male members of the family seeking treatment while women’s health is ignored. Another issue is that many of the women suffering from AKI were left at their parents’ homes by their husbands. If their fathers were alive they would make efforts to bring their daughters for follow up visits. In the case where fathers had died and brothers were married, they were reluctant to bring their sisters for treatment as their wives believed that this was the responsibility of the patients’ husbands, not of their brothers. Accordingly, CKD as an outcome was also highest in the obstetrical category. Among the 280 women in this group developing CKD, 213 showed acute cortical necrosis (ACN) either on radiological findings, or histopathology, or both. Histopathology has shown both varieties, focal in some and diffuses patterns of ACN in others.

Acute gastroenteritis/diarrheal illness still remains on top of our medical causes of AKI though in recent years (group IV) malarial infections and toxic rhabdomyolysis mainly resulting from PPD poisoning has superseded this cause (Table-II). We have seen a change in trends of rhabdomyolysis during the last five years of this study. Previously this particular group used to include more crush syndrome and exercise induced rhabdomyolysis; now we see more of toxic rhabdomyolysis resulting from PPD ingestion.^23^ This is different from regional studies, where cases have been reported with multiple wasp stings, alcohol binge drinking, and a small number of cases from PPD poisoning and with dengue infection.^17^

Malaria; according to a 2018 WHO report, affects 228 million people worldwide, causing death to 405,000 people, mostly residing in the tropics / or mainly in LICs and LMICs.^34^ In malarial AKI we have seen that in the later period of the study, AKI caused by vivax malaria was increasing but predictors of mortality remain unchanged over the years.^21^,^35^ Another institution in the country has reported similar findings of increasing vivax malaria as cause of AKI.^36^ Overall reported contribution of malaria to AKI is 2-39% in different populations.^37^ In comparison to tropical region smaller numbers are reported from western countries.

AKI resulting from dengue infection has also been reported in Pakistan where AKI was found in 13.3% (71/532) of all dengue cases studied. Approximately two-thirds (64.8%) of these patients had mild AKI and a third (35.2%) had moderate to severe AKI.^38^ In our study we have recorded dengue in patients who came with AKI (as this is tertiary care renal facility), so prevalence of AKI with dengue infection cannot be commented in this scenario.^22^

While snake bite causing AKI has been reported frequently from tropical countries over the last few decades some older studies discuss mechanisms of injury in detail.^39^ From our institution we have also published details of presentation and outcome in this particular group of medical AKI.^25^

Scorpion sting causing AKI has been reported less frequently, though health hazards associated with scorpion sting which is more common in Middle East, Iran, Latin America, South America, North Africa, India and Pakistan, has been reported. 40 In a previous publication from our institution mechanisms of kidney injury has been discussed in some details.^26^

Surgical cause related to bilateral obstruction was included only in Group-I in this study, the reason being that with expansion of services and institutional premises, these cases were followed by urology counter members of our team. Keeping long term follow up for them became very difficult in a scenario where all data had been collected manually, requiring us to go through each and every case record or look at patients physically. In the majority of patients, the cause of obstruction was bilateral ureteric or renal stones or stone causing obstruction to the solitary functioning kidney. In some patients it was pelviureteric junction obstruction. There were a few cases of prostatic enlargement in males, cervical cancers in females or pelvic mass in both genders. (Table-IV).

Complete renal recovery in medical AKI over the years has shown small variation from group I to IV, similarly there was little decline in deaths during acute phase of illness over different groups. In obstetrical AKI number of women developing CKD is higher when compared to other groups. Different factors which revealed significance with mortality either in isolation or in combination are given in Table-V.

## CONCLUSION

In this part of world, we still see AKI resulting from many preventable causes. This study addresses causes of CA-AKI from a LMIC for first time in such a large population. It also reflects that even when patients arriving with advanced renal dysfunction reaching to a facility where proper renal care can be provided it may give acceptable outcome in terms of patient survival and to variable extent renal survival as well. Provision of health care facility at door step might resolve issues of outcome of many partially recovered group of patients.
